# Methadone Dose and Patient-Directed Discharge in Hospitalized Patients With Opioid Use Disorder

**DOI:** 10.1001/jamanetworkopen.2026.3439

**Published:** 2026-03-25

**Authors:** Rebecca R. Meredith, William M. Garneau, Kenneth A. Feder, Megan E. Buresh

**Affiliations:** 1 Division of Hospital Medicine, Department of Medicine, Johns Hopkins Hospital, Baltimore, Maryland; 2 Department of Mental Health, Johns Hopkins Bloomberg School of Public Health, Baltimore, Maryland; 3 Division of Addiction Medicine, Department of Medicine, Johns Hopkins School of Medicine, Baltimore, Maryland

## Abstract

**Importance:**

Patient-directed discharge (PDD), when patients leave the hospital prior to completing recommended medical treatment, is associated with increased morbidity and mortality and occurs in 10% to 20% of hospitalizations for patients with opioid use disorder (OUD). Understanding risk factors associated with PDD is essential to improving outcomes for this population.

**Objective:**

To investigate whether hospitalized patients with OUD who received higher doses of methadone during the first 24, 48, and 72 hours after first contact with the emergency department had decreased odds of PDD.

**Design, Setting, and Participants:**

This retrospective observational cohort study was conducted at a single academic health center in the northeastern US during the fentanyl era (July 1, 2019, to June 30, 2022). Hospitalized adults with OUD and without methadone listed in their medication history who received methadone during the first 72 hours were included. Data were analyzed from April 2025 through February 2026.

**Exposures:**

Cumulative dose of methadone received for patients 24, 48, and 72 hours after initial evaluation in the emergency department.

**Main Outcomes and Measures:**

PDD by 48, 72, or 96 hours or ever, as indicated by discharge disposition in the patient electronic health record.

**Results:**

A total of 554 patients were included in the study. For analysis, participants were separated into cohorts based on cumulative dose by 24 hours (325 patients), 48 hours (488 patients), and 72 hours (454 patients) after presentation to the emergency department, with the main analysis among patients in the 24-hour cohort. Among 325 patients (184 male [56.6%]; median [IQR] age, 49.0 [36.0-59.0] years) receiving methadone within 24 hours of presentation to the emergency department, the incidence of PDD was 45 patients (13.8%). In an adjusted logistic regression model, each additional 10 mg of methadone in the first 24 hours was associated with lower odds of PDD (adjusted odds ratio [aOR], 0.71; 95% CI, 0.44-0.98) at 48 hours. Results were similar for PDD at 72 hours (aOR, 0.68; 95% CI, 0.50-0.85), 96 hours (aOR, 0.72; 95% CI, 0.56-0.88), or ever (aOR, 0.79; 95% CI, 0.67-0.91) in the 24-hour cohort and qualitatively similar but with smaller decreases in odds or nonsignificant outcomes for cumulative methadone dose in the 48-hour cohort (eg, PDD at 96 hours: aOR, 0.91; 95% CI, 0.82-0.99) and nonsignificant outcomes in the 72-hour cohort (eg, PDD at 96 hours: aOR, 0.98; 95% CI, 0.89-1.06).

**Conclusions and Relevance:**

In this study, higher cumulative doses of methadone during the first 48 hours of care were associated with substantial reductions in the incidence of PDD. These findings suggest that early and adequate treatment of withdrawal with methadone may be associated with reduced PDD among hospitalized patients with OUD in the fentanyl era.

## Key Points

**Question:**

Among hospitalized patients with opioid use disorder, is there an association between the early cumulative dose of methadone and risk of patient-directed discharge?

**Findings:**

In this cohort study of 554 adults with opioid use disorder who were admitted to the hospital and received methadone during the first 72 hours, there was a decreased risk of patient-directed discharge with increased methadone dose given during the first 24 hours of care in an adjusted analysis.

**Meaning:**

These findings suggest that in the fentanyl era, early treatment with a higher cumulative dose of methadone may be associated with reduced patient-directed discharge.

## Teaser

This cohort study investigates the association of higher early doses of methadone with odds of patient-directed discharge among hospitalized patients with opioid use disorder.

## Introduction

The US continues to grapple with an opioid crisis. In 2024, approximately 48 000 people in the US died from an opioid-involved overdose. Hospitalization rates for people with opioid use disorder (OUD) increased by 64% from 2006 to 2016.^1^ Hospitalizations are a reachable moment when patients can start medications for opioid use disorder (MOUD), methadone and buprenorphine.^2^ However, only 15% to 20% of patients with OUD receive MOUD during hospitalization.^3,4^ Additionally, approximately 10% to 20% of hospitalizations for patients with OUD end in a patient-directed discharge (PDD), defined as leaving the hospital prior to completing recommended treatment, compared with 1% to 2% for patients without OUD.^5,6^ PDD leads to increased readmission, mortality, and higher health care costs.^7,8^ Patients with OUD often cite untreated pain and withdrawal as contributing to their decisions to leave the hospital by PDD.^7,9^

Fentanyl is prevalent in the local drug supply and is involved in more than 70% of fatal drug overdoses in Baltimore.^10^ MOUD initiation can be more complex in persons using fentanyl due to the severity of withdrawal given higher opioid tolerance and increased risk of precipitated withdrawal with buprenorphine due to fentanyl’s lipophilicity.^11^ There is preliminary evidence that inpatient treatment may be associated with PDD among patients with OUD. A large retrospective cohort study^12^ demonstrated low rates of PDD in hospitalized patients treated with MOUD. Small case series have additionally illustrated low rates of PDD with novel strategies, including rapid methadone titration and the use of short-acting opioid agonists.^13,14,15,16^ However, there is a lack of work examining the association of MOUD dose and timing with PDD while controlling for factors such as prior MOUD maintenance and concurrent opioid administration during hospitalization.^12,17,18^

In this study, we investigated the association between the timing and dosing of methadone and subsequent risk for PDD among hospitalized patients with OUD. We focused on methadone because this medication can be given in the context of recent fentanyl administration without precipitating withdrawal and because of increasing preference for methadone among patients with fentanyl dependence.^19^ Our purpose was to examine the association between initial methadone treatment and subsequent PDD.

## Methods

### Study Design

We performed a retrospective observational cohort study of adult patients with OUD hospitalized at the Johns Hopkins Hospital between July 1, 2019, and June 30, 2022. Source data, including patient demographics, home medication lists (eg, medications noted on admission), episode of care (eg, date and time of first emergency department [ED] contact), clinical results (eg, Clinical Opiate Withdrawal Scale [COWS] score), clinical care (eg, date and time of treatment with methadone), and patient disposition (eg, PDD), were bulk extracted from the relevant encounter within applicable tables in the Epic electronic health record. Data extraction was iteratively validated by the study team (R.R.M. and W.M.G.) and data analyst to ensure validity. For missing data, we performed manual electronic health record review. The study was approved by the Johns Hopkins School of Medicine Institutional Review Board under a waiver of informed consent due to impracticability. Results are reported in line with the Strengthening the Reporting of Observational Studies in Epidemiology (STROBE) reporting guideline.

### Setting

Johns Hopkins Hospital is a 1215-bed academic hospital located in Baltimore, Maryland. An inpatient addiction medicine service was launched in July 2020. Historically, clinicians could order a maximum initial methadone dose of 20 mg or less for patients not enrolled in an opioid treatment program. In 2022, hospital policy was modified to allow an initial methadone dose of 40 mg or less without addiction medicine approval. Higher initial doses could be ordered (no dose limit) with approval or upon confirming treatment with an opioid treatment program. During the study period, methadone was uptitrated in 10-mg intervals and reassessed daily.

### Participants

Eligibility criteria included adults aged 18 years or older admitted for inpatient hospitalization from the ED for any reason; an *International Statistical Classification of Diseases, Tenth Revision, Clinical Modification *(*ICD-10-CM*) code consistent with OUD in prior medical history or active problem list (F11.XX) during the past 12 months; receipt of methadone during the first 72 hours after first ED contact; and not receiving methadone maintenance at admission, defined as the absence of methadone on the home medication list. The final criterion was added given that patients receiving methadone maintenance often receive higher baseline doses and are a distinct clinical population from patients newly starting methadone. For patients with multiple admissions during the study period, only the first admission was included. Participants were excluded if they had a missing value on a covariate included in the multivariable analysis. Persons could receive buprenorphine in addition to methadone.

The cohort was identified by searching for all hospital encounters among adults with an OUD *ICD-10-CM* diagnosis code between July 1, 2019, and June 30, 2022. We filtered for the first encounter for each distinct patient, excluded any patient with methadone listed on the home medication list, and included persons who received methadone during the first 72 hours of admission. Finally, we excluded patients who did not present to the main ED (eg, those who presented to labor and delivery) and those with missing covariates used in the analysis.

**Figure 1.**
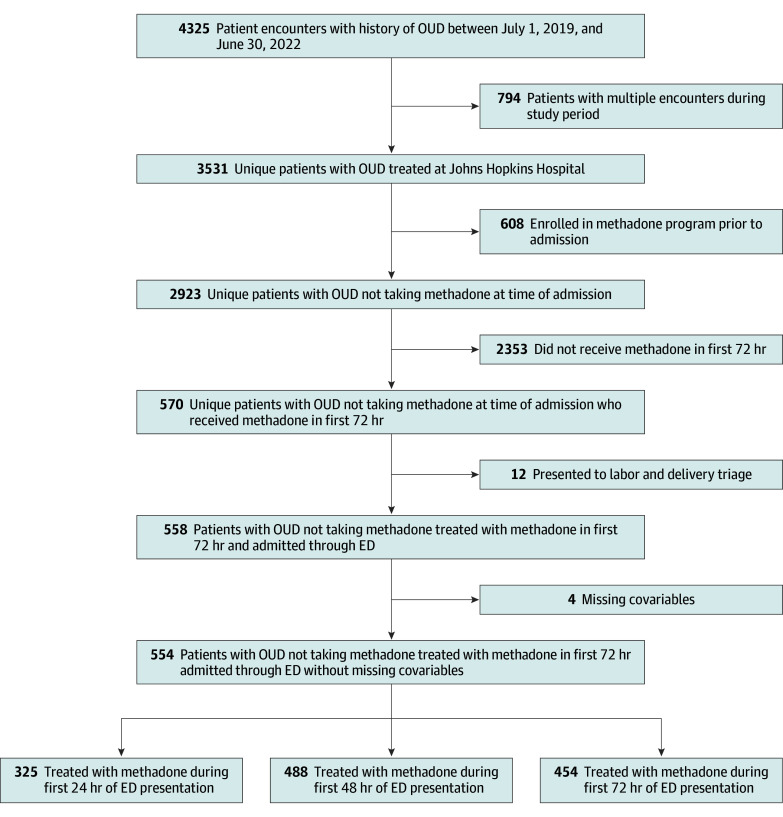
Study Flowchart ED indicates emergency department; OUD, opioid use disorder.

Using this sample, we constructed cohorts of patients admitted to the hospital for at least 24, 48, and 72 hours, examining the association of cumulative methadone dose with PDD. Time zero was defined as the patient’s first contact with ED. Patients who received methadone from hours 0 to 24 from ED triage and did not have PDD prior to 24 hours were included in the 24-hour cohort; patients who received methadone from hours 0 to 48 and did not have PDD prior to 48 hours were included in the 48-hour cohort, and patients who received methadone from hours 0 to 72 and did not have PDD prior to 72 hours were included in the 72-hour cohort. The inclusion process is illustrated in Figure 1.

### Exposure and Outcome

Exposures of interest were cumulative doses of oral methadone received in the first 24, first 48, and first 72 hours. Dosages were analyzed as a continuous variable measured in 10-mg increments. Treatment with intravenous methadone was not included owing to differences in pharmacokinetics and bioavailability between routes of administration. For descriptive tables, methadone dosage was also categorized by tertile. For continuous variables, Shapiro-Wilk normality testing was performed; median values are reported for nonnormally distributed variables.

The primary outcome of interest was PDD, which was defined as a hospital discharge disposition of *against medical advice* or *AWOL*. We examined this outcome at 4 times: at 48 hours, 72 hours, and 96 hours from time of ED triage and at any point during hospitalization (*ever*).

### Potential Confounders

Potential confounding variables were selected a priori based on the literature.^20^ The multivariable model adjusted for age, sex, race, insurance, smoking, ED length of stay, initial COWS score, addiction consultation order, treatment with opioid patient-controlled analgesia (PCA), and non-MOUD opioid analgesia during relevant time blocks. Non-MOUD opioid analgesia was captured as a continuous variable in milligram of morphine equivalents. Treatment with PCA was dichotomized given that precise doses were not available. Race and ethnicity were self-reported and extracted in bulk from electronic health records (ethnicity was not adjusted in the model). Race options were American Indian or Alaska Native, Asian (includes Asian, Asian Indian, Korean, Vietnamese, and other Asian), Black or African American, Native Hawaiian or Other Pacific Islander (includes Native Hawaiian or Other Pacific Islander and Guamanian or Chamorro), White, and other race. Ethnicity options were Hispanic or Latino (includes Chicano/a, Cuban, Mexican, Mexican American, Puerto Rican, and another Hispanic Latino or Spanish origin) and not Hispanic or Latino.

### Statistical Analysis

For each cohort, we estimated the cumulative incidence of each PDD outcome among the full cohort and within each tertile of cumulative methadone exposure. As an exploratory analysis, we also graphically display a locally estimated scatterplot smoothing (LOESS) plot showing the association of methadone dose with PDD. For our main analysis, we estimated crude odds ratios (ORs) and adjusted ORs (aORs) for associations of each cumulative methadone dose with each PDD outcome using univariable and multivariable logistic regression models, respectively. Exponentiated coefficients are interpreted as the relative change in the odds of PDD per 10-mg increase in cumulative methadone dose during the relevant time window. For all estimates, we present 95% profile CIs; the statistical significance of each OR was estimated at the *P* < .05 level using likelihood ratio tests; 2-sided *P* values were used. Abstracted data were reviewed in a HIPAA-compliant virtual computing environment using Excel for Microsoft 365 MSO version 2511 (Microsoft Corp),^21^ and data were analyzed in R statistical software version 4.5.0 (R Project for Statistical Computing).^22^ Data were analyzed from April 2025 through February 2026.

## Results

There were 4325 total hospital encounters among adults with an OUD *ICD-10-CM* diagnosis code between July 1, 2019, and June 30, 2022. Among these 4325 encounters, there were 3531 unique patients identified, of whom 2923 individuals did not have methadone listed on their home medication list. After exclusion of patients who did not receive methadone during the first 72 hours of admission, did not present to the ED, or were missing values on covariates used in the analysis, our final sample included 554 patients.

Using this sample, we constructed cohorts of patients admitted to the hospital for at least 24, 48, and 72 hours, examining the association of cumulative methadone dose with PDD, with the main analysis in the 24-hour cohort. The sample size for each cohort was 325 patients for the 24-hour cohort (median [IQR] age, 49.0 [36.0-59.0] years; 184 male [56.6%]; 1 Asian [0.3%], 179 Black [55.1%], and 139 White [42.8%]; 2 Hispanic [0.6%]), 488 patients for the 48-hour cohort (median [IQR] age, 49.5 [37.0-59.0] years; 282 male [57.8%]; 2 Asian [0.4%], 277 Black [56.8%], and 198 White [40.6%]; 4 Hispanic [0.8%]), and 454 patients for the 72-hour cohort (median [IQR] age, 50.0 [37.0-59.0] years; 261 male [57.5%]; 2 Asian [0.4%], 262 Black [57.7%], and 181 White [39.9%]; 4 Hispanic [0.9%]). Characteristics for the 24-hour cohort are presented in Table 1; 48-hour and 72-hour cohort baseline characteristics are presented in eTable 1 and eTable 2 in Supplement 1.

**Table 1.** Demographics and Clinical Characteristics of Patients Who Received Methadone Within 24 Hours of ED Presentation

Characteristic	Patients, No. (%)^a^			
	Low-dose methadone (<30 mg) (n = 128)	Medium-dose methadone (30-60 mg) (n = 93)	High-dose methadone (>60 mg) (n = 104)	Overall (N = 325)
Age, median (range), y	44.0 (23.0-71.0)	52.0 (23.0-78.0)	51.0 (26.0-82.0)	49.0 (23.0-82.0)
Sex				
Female	62 (48.4)	43 (46.2)	36 (34.6)	141 (43.4)
Male	66 (51.6)	50 (53.8)	68 (65.4)	184 (56.6)
Race				
American Indian or Alaska Native	0	0	0	0
Asian	1 (0.8)	0	0	1 (0.3)
Black or African American	69 (53.9)	53 (57.0)	57 (54.8)	179 (55.1)
Native Hawaiian or Other Pacific Islander	0	0	0	0
White	57 (44.5)	36 (38.7)	46 (44.2)	139 (42.8)
Other^b^	1 (0.8)	4 (4.3)	1 (1.0)	6 (1.8)
Ethnicity				
Hispanic or Latino	1 (0.8)	1 (1.1)	0	2 (0.6)
Not Hispanic or Latino	127 (99.2)	91 (97.8)	104 (100)	322 (99.1)
Other non-Hispanic^c^	0	1 (1.1)	0	1 (0.3)
Insurance type				
Medicare	19 (14.8)	18 (19.4)	19 (18.3)	56 (17.2)
Medicaid	99 (77.3)	67 (72.0)	69 (66.3)	235 (72.3)
Military	0	0	0	0
Uninsured	0	0	0	0
Commercial	10 (7.8)	8 (8.6)	16 (15.4)	34 (10.5)
Hospital level of care at admission				
Intensive care unit	3 (2.3)	1 (1.1)	2 (1.9)	6 (1.8)
Progressive care unit	2 (1.6)	3 (3.2)	1 (1.0)	6 (1.8)
Monitored floor	53 (41.4)	38 (40.9)	35 (33.7)	126 (38.8)
Nonmonitored floor	68 (53.1)	47 (50.5)	65 (62.5)	180 (55.4)
Other^d^	2 (1.6)	4 (4.4)	1 (1.0)	7 (2.1)
Admitting service				
Medicine	111 (86.7)	73 (78.5)	84 (80.8)	268 (82.5)
Surgery	11 (8.6)	7 (7.5)	11 (10.6)	29 (8.9)
Obstetrics	1 (0.8)	6 (6.5)	1 (1.0)	8 (2.5)
Pediatrics	0	0	0	0
Neurology	1 (0.8)	3 (3.2)	4 (3.8)	8 (2.5)
Other^e^	4 (3.1)	4 (4.3)	4 (3.8)	12 (3.7)
Current or former smoker	122 (95.3)	88 (94.6)	96 (92.3)	306 (94.2)
ED length of stay, median (range), h	13.7 (0-96.7)	11.5 (0.2-48.7)	14.6 (0-46.2)	13.1 (0-96.7)
COWS ordered during admission	87 (68.0)	47 (50.5)	23 (22.1)	157 (48.3)
Initial COWS score				
Not performed	41 (32.0)	46 (49.5)	81 (77.9)	168 (51.7)
0-2	31 (24.2)	17 (18.3)	11 (10.6)	59 (18.2)
2-5	27 (21.1)	14 (15.1)	9 (8.7)	50 (15.4)
>5	29 (22.7)	16 (17.2)	3 (2.9)	48 (14.8)
Addiction consultation during admission	49 (38.3)	23 (24.7)	14 (13.5)	86 (26.5)
Opioid PCA during admission	19 (14.8)	11 (11.8)	5 (4.8)	35 (10.8)
MME (non-MOUD) in first 24 h, median (range), h	6.00 (0-286.00)	0 (0-750.00)	0 (0-173.00)	0 (0-750.00)

Abbreviations: ED, emergency department; COWS, clinical opiate withdrawal scale; PCA, patient-controlled analgesia; MME, morphine milligram equivalent; MOUD, medications for opioid use disorder.

^a^ Demographic and clinical characteristics of included patients are given stratified by the dose of methadone received within the first 24 hours of presentation to the ED.

^b^ Includes other and choose not to disclose.

^c^ Includes other, choose not to disclose, and unknown.

^d^ Includes other and missing.

^e^ Includes emergency medicine and missing.

Among patients in the 24-hour cohort, PDD occurred in 14 patients (4.3%) at 48 hours, 26 patients (8.0%) at 72 hours, 31 patients (9.5%) at 96 hours, and 45 patients (13.8%) ever during admission. Among patients in the 48-hour cohort, PDD occurred in 17 patients (3.5%) at 72 hours, 27 patients (5.5%) at 96 hours, and 48 patients (9.8%) ever during admission. Finally, among patients in the 72-hour cohort, PDD occurred in 10 patients (2.2%) at 96 hours and 32 patients (7.0%) ever during admission (Table 2).

**Table 2.** Incidence of PDD Throughout Hospitalization

Cohort	Patients with PDD, No. (%)^a^			
	Low-dose methadone	Medium-dose methadone	High-dose methadone	Overall
24-h Cohort^b^				
Patients, No.	128	93	104	325
PDD at 48 h	10 (7.8)	4 (4.3)	0	14 (4.3)
PDD at 72 h	18 (14.1)	7 (7.5)	1 (1.0)	26 (8.0)
PDD at 96 h	22 (17.2)	7 (7.5)	2 (1.9)	31 (9.5)
PDD ever	30 (23.4)	10 (10.8)	5 (4.8)	45 (13.8)
48-h Cohort^b^				
Patients, No.	169	157	162	488
PDD at 72 h	6 (3.6)	9 (5.7)	2 (1.2)	17 (3.5)
PDD at 96 h	13 (7.7)	11 (7.0)	3 (1.9)	27 (5.5)
PDD ever	23 (13.6)	17 (10.8)	8 (4.9)	48 (9.8)
72-h Cohort^b^				
Patients, No.	162	144	148	454
PDD at 96 h	7 (4.3)	2 (1.4)	1 (0.7)	10 (2.2)
PDD ever	13 (8.0)	15 (10.4)	4 (2.7)	32 (7.0)

Abbreviation: PDD, patient directed discharge.

^a^ The distribution of PDDs during the course of hospitalization (within 24, 48, 72, and 96 hours of emergency department presentation or ever during hospitalization) is shown stratified by methadone dosing tertile. The distribution of PDD is shown for 3 cohorts defined by total methadone dose received within 24 hours, 48 hours, and 72 hours of presentation to the ED.

^b^ Methadone doses were defined as less than 30 mg for low, 30 to 60 mg for medium, and more than 60 mg for high in the 24-hour cohort; less than 50 mg for low, 50 to 105 mg for medium, and more than 105 mg for high in the 48-hour cohort; and less than 80 mg for low, 80 to 170 mg for medium, and more than 170 mg for high in the 72-hour cohort.

In univariable analysis of the 24-hour cohort, the odds of PDD at 48 hours decreased for each 10-mg increase in methadone dose (OR, 0.67; 95% CI, 0.47-0.87). This association was also present for PDD at 72 hours (OR, 0.68; 95% CI, 0.53-0.83), at 96 hours (OR, 0.71; 95% CI, 0.58-0.85), and ever (OR, 0.77; 95% CI, 0.66-0.87). Univariable analysis of OR of PDD by dosage was also performed for the 48-hour cohort with similar findings; the findings were not statistically significant for the 72-hour cohort (Table 3).

**Table 3.** Association of Methadone Dose With Odds of PDD

Cohort	PDD, unadjusted OR (95% CI)^a^	*P* value	PDD, adjusted OR (95% CI)^b^	*P* value
24-h Cohort				
PDD at 48 h	0.67 (0.47-0.87)	.001	0.71 (0.44-0.98)	.03
PDD at 72 h	0.68 (0.53-0.83)	<.001	0.68 (0.50-0.85)	<.001
PDD at 96 h	0.71 (0.58-0.85)	<.001	0.72 (0.56-0.88)	<.001
PDD ever	0.77 (0.66-0.87)	<.001	0.79 (0.67-0.91)	.001
48-h Cohort				
PDD at 72 h	0.91 (0.81-1.00)	.046	0.91 (0.80-1.01)	.08
PDD at 96 h	0.90 (0.81-0.97	.005	0.91 (0.82-0.99)	.03
PDD ever	0.92 (0.87-0.98)	.005	0.94 (0.87-0.99)	.03
72-h Cohort				
PDD at 96 h	0.95 (0.87-1.03)	.23	0.98 (0.89-1.06)	.70
PDD ever	0.97 (0.92-1.01)	.11	0.98 (0.93-1.03)	.37

Abbreviations: OR, odds ratio; PDD, patient-directed discharge.

^a^ Odds of patient-directed discharge with 10-mg increase in methadone dose, unadjusted.

^b^ Odds of patient-directed discharge with 10-mg increase in methadone dose, adjusted for age, race, gender, emergency department length of stay (continuous, in hours), ever smoker, opioid patient-controlled analgesia (yes or no), addiction consult (yes or no), initial Clinical Opiate Withdrawal Scale score (no score, 0-2, 2-5, or >5), and total treatment other than medications for opioid use disorder in morphine milligram equivalents during the first 24, 48, or 72 hours.

**Figure 2.**
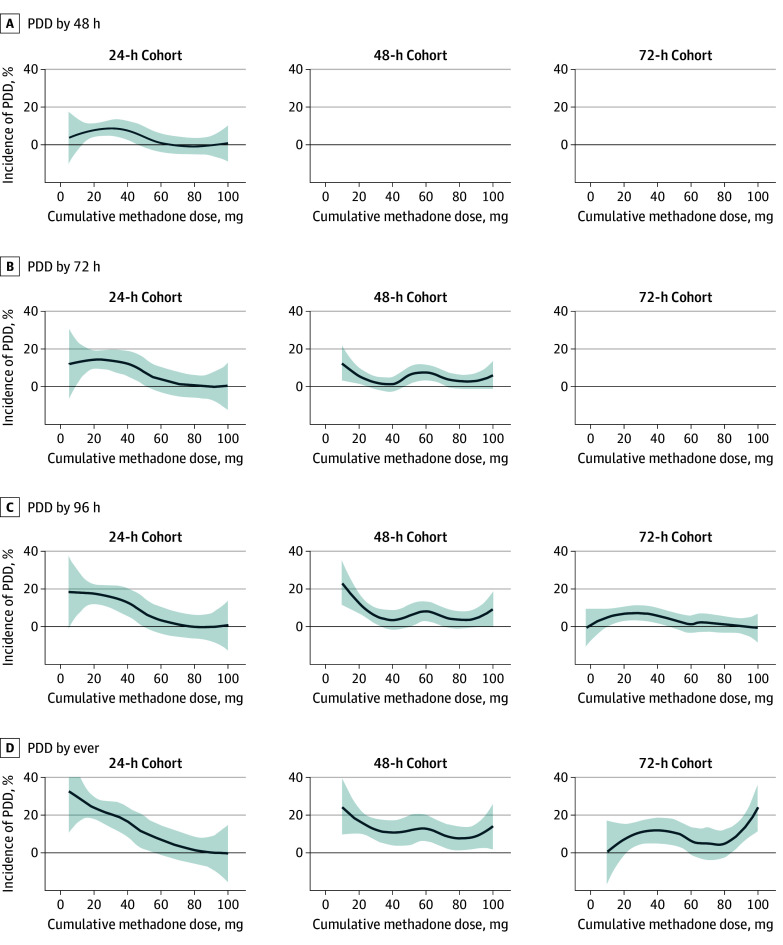
Line Graph of Incidence of Patient-Directed Discharge (PDD)

In multivariable analysis, higher methadone dose was associated with lower odds of PDD at 48 hours for the 24-hour cohort (aOR, 0.71; 95% CI, 0.44-0.98). This association was also present for PDD at 72 hours (aOR, 0.68; 95% CI, 0.50-0.85), at 96 hours (aOR, 0.72; 95% CI, 0.56-0.88), and ever (aOR, 0.79; 95% CI, 0.67-0.91) in this cohort. Multivariable analysis of aOR of PDD by dosage was also performed for groups analyzed at 48 hours and 72 hours with similar findings, although decreases in odds were smaller or nonsignificant for some outcomes in the 48-hour cohort (eg, PDD at 96 hours: aOR, 0.91; 95% CI, 0.82-0.99) and nonsignificant for all outcomes in the 72-hour cohort (eg, PDD at 96 hours: aOR, 0.98; 95% CI, 0.89-1.06) (Table 3). Covariable distributions as used in the multivariable model are included in eTable 3 in Supplement 1. A graphic representation of the incidence of PDD for the 24-hour, 48-hour, and 72-hour analytic groups by cumulative methadone dose is presented using a LOESS plot in Figure 2.

## Discussion

In this retrospective cohort study of 325 hospitalized patients, increases in methadone dose during early hospitalization were associated with decreased odds of PDD. Each 10-mg increase in methadone received in the first 24 hours of the hospital course was associated with a reduction in the odds of PDD by 48 hours (aOR, 0.71). Our second finding was that the total dosage of methadone received in the first 24 hours after presentation to the ED was distinctly associated with a decrease in odds of subsequent PDD. These findings together are consistent with the hypothesis that prioritizing earlier and more aggressive treatment of withdrawal with methadone may be associated with reduced PDD. Because PDD is associated with worse patient outcomes across a range of conditions,^8^ providing earlier, higher doses of methadone may be associated with improved patient outcomes and care quality. This is an important area for future research.

The association between MOUD and PDD has been previously investigated. Several single-center studies found that methadone and buprenorphine may be associated with protection against PDD; however, these studies were limited by small sample sizes and lack of dose- and timing-specific data.^23,24,25^ A large retrospective cohort study in 2023^12^ that included 127 158 hospitalizations across the US found that receipt of early MOUD (defined as any buprenorphine or methadone during the first 2 days of hospitalization) was associated with a decrease in the risk of PDD (aOR, 0.73). Our study further builds on these previous findings by focusing on the benefit associated with methadone in new-start MOUD therapy specifically; this was done by controlling for receipt of non-MOUD opioids during hospitalization and, most importantly, by identifying benefits associated with early administration of higher doses.

A 2021 retrospective cohort study^17^ that included more than 1000 hospital encounters also demonstrated an association of MOUD with protection against PDD and additionally included more detailed data on dosing. In that study, dosing was split into 4 categories (one-time dose, dose reduction, dose escalation, and same as initial dose). The authors found that among patients who received methadone, a higher proportion of patients discharged as PDD received lower initial doses or had a dose reduction before discharge. However, specific dosing and timing of MOUD and non-MOUD opioids were not reported.^17^

Numerous qualitative studies have reported that patients cite undertreated withdrawal and pain as significant factors in their decision to leave the hospital.^9,26^ Methadone is a highly potent full μ-opioid agonist and can be used to partially offset the reduction in total opioids that patients with OUD may experience during the transition from using illicitly manufactured fentanyl to receiving treatment in the inpatient setting. In Baltimore, fentanyl first emerged in 2013 and has been involved in most opioid-related overdoses since 2015.^27^ Other authors in fentanyl-predominant locales have reported improved response associated with high-dose protocols for short-acting opioids.^15,28^

While there is no definitive consensus on methadone dosing in the fentanyl era, current guidelines suggest a minimum dosing of 60 to 160 mg, with higher doses associated with higher retention in treatment.^29^ Given methadone’s long half-life and the concern for eventual oversedation, methadone is titrated gradually in 10- to 20-mg intervals every 3 to 7 days, with therapeutic dosing often taking weeks.^30^ However, these guidelines were adopted for outpatient use during the heroin era and may not be adequate for acute care now.^13^

Retrospective studies have demonstrated low incidence of adverse outcomes with rapid methadone titration in the hospital setting.^13,14,16,31,32,33^ A total of 247 patients were included in 4 unique 2023 retrospective case series examining the safety of methadone initiation and dose escalation over 5 to 7 days during hospitalization.^13,14,32,33^ In these studies, the mean starting dose ranged between 30 and 40 mg. It took a mean of 5 to 7 days to achieve the mean maximum dose, which ranged from 62 mg to 76 mg. There were approximately 47 reports of sedation (19%), but only 2 were deemed serious. A total of 42 unique patients were included in 2 separate rapid titration protocols, which achieved maximum dosing of 80 to 100 mg over 3 days from a starting dose of 60 mg, without serious adverse events.^16,31^

Despite the well-established benefits associated with hospital MOUD, rates of MOUD initiation in hospitalized patients remain low.^3,4^ Several barriers contribute to this. These include lack of addiction medicine training among clinicians, hospital policies that limit initial methadone dose without an addiction medicine consult, general stigma in health care directed toward patients with OUD, and historical segregation of methadone from general medical treatment. Furthermore, the pharmacology and pharmacokinetics of methadone may lead to concerns about dose stacking and oversedation.^34^

Our findings suggest that when patients with OUD are treated with methadone while in an inpatient setting, higher initial methadone doses are associated with decreased risk for PDD, with particularly large decreases in risk for doses in the first 24 hours of hospitalization. Developing evidence to support new standards for treating OUD in the hospital that reflect realities of the fentanyl era are of critical importance in addressing the opioid epidemic.

### Limitations

Limitations of this study include the use of *ICD-10-CM* codes to identify patients in the cohort given that these codes have limited specificity and may mischaracterize patients who may not meet criteria for active OUD. Our regression models implicitly assumed that there was a linear association between methadone dose and the log odds of each PDD outcome. We were unable to analyze methadone dose using ordinal dummy variables owing to small sample sizes of PDD in high-dose groups. This is an important topic for future research in a larger sample. In our study, the use of a 12-month lookback period for OUD-related *ICD-10-CM* diagnoses was likely to have improved sensitivity, possibly at the expense of specificity with regards to active OUD; however, in further refining the cohort to include only patients treated with methadone during hospitalization, we were able to achieve high sensitivity and specificity for the cohort of interest. This study is not a randomized clinical trial, and we did not have admitting diagnoses or full comorbidities and were unable to adjust for all potential confounders. In particular, we could not account for stimulant use disorder, a known risk factor for PDD in this population.^35^ While we attempted to exclude patients receiving methadone prior to admission using the medication history report, it is possible that some patients who were on maintenance treatment were included due to the medication list being inaccurate. In our study, we looked at cumulative methadone dose at distinct times and not specific methadone titration protocols, so we cannot comment on the association of dose escalation with outcomes. We considered comparing patients who received methadone with a control group of patients who did not receive any MOUD. However, identifying an appropriate set of patients for a control group proved challenging. This is because it is not easy to identify which patients require methadone treatment from *ICD-10-CM* codes alone given that a patient OUD diagnosis may be outdated and patients may no longer require methadone treatment when admitted to the hospital. For this study focused on methadone dose, we felt a better choice was to restrict the analysis to participants who received methadone given that this is a population for whom our research question is clearly relevant. Comparing patients who receive MOUD with patients who do not receive it may be the focus of a future study.

## Conclusions

This cohort study found that earlier and higher doses of methadone were associated with substantial decreases in odds of PDD. While several retrospective case series have demonstrated low rates of adverse events with rapid inpatient methadone titration, larger multicenter studies and randomized clinical trials are needed to investigate the safety and efficacy of rapid inpatient methadone titration for the treatment of acute withdrawal. Finally, in the era of fentanyl, it is imperative that we work toward educating hospitalists and hospital systems on the use of methadone for the management of acute withdrawal for hospitalized patients with OUD.

## Notes

Supplement 1. eTable 1. Demographics and clinical characteristics of patients who received methadone within 48 h of ED presentation eTable 2. Demographics and clinical characteristics of patients who received methadone within 72 h of ED presentation eTable 3. Covariable distributions as used in multivariable model for PDD risk for 24-h, 48-h, and 72-h cohorts

Supplement 2.
Data Sharing Statement
