# Supplementary material for: Methadone Dose and Patient-Directed Discharge in Hospitalized Patients With Opioid Use Disorder
Source: JAMA Netw Open. 2026 Mar 25;9(3):e263439. doi: 10.1001/jamanetworkopen.2026.3439 (PMC13019229; doi:10.1001/jamanetworkopen.2026.3439)
Supplement: Supplement 2. — Data Sharing Statement [file jamanetwopen-e263439-s002.pdf]

## Data Sharing Statement

Meredith. Methadone Dose and Patient-Directed Discharge in Hospitalized Patients With Opioid Use Disorder. *JAMA Netw Open*. Published online March 25, 2026. doi:10.1001/jamanetworkopen.2026.3439

**Data**
**Data available:** Yes
**Data types:** Deidentified participant data
**How to access data:** william.garneau@jhmi.edu
**When available:** With publication

**Supporting Documents**
**Document types:** Statistical/analytic code
**How to access documents:** william.garneau@jhmi.edu
**When available:** With publication

**Additional Information**
**Who can access the data:** Researchers whose proposed use of the data has been approved
**Types of analyses:** Data will be provided for verification and reproducibility
**Mechanisms of data availability:** With investigator support after signed data access agreement
**Any additional restrictions:** Limited data set for verification purposes with approval from IRB
